# Supplementary material for: The Gaussian-linear hidden Markov model: A Python package
Source: Imaging Neurosci (Camb). 2025 Feb 3;3:imag_a_00460. doi: 10.1162/imag_a_00460 (PMC12319829; doi:10.1162/imag_a_00460)
Supplement: Supplementary Material [file imag_a_00460-supp.pdf]

Supplemental information

Data specifications

ECoG, monkey brain-computer interface data. This dataset was pre-collected and made publicly available. Data were first presented in (Chao et al., 2010). The ECoG signals were recorded at a sampling rate of 1 kHz per channel, for a total of thirty-two electrodes implanted in the right hemisphere, and band-pass filtered from 0.3 to 500 Hz. We considered a time-frequency representation of the ECoG signals containing 1600 variables (32 electrodes, 10 frequency bins, and 5 time lags), from which we extracted 10 principal components, and downsampled to 250Hz. The monkey's movements were captured at a sampling rate of 120 Hz, and upsampled to 250Hz to match the sampling frequency of the brain data. We used data from three different monkeys, with one continuous scanning session of 2991, 1499 and 1499 time points after sampling, respectively.

Magnetoencephalography, visual-memory task. Neuromagnetic data were acquired using a whole-head VectorView system (204 planar gradiometers, 102 magnetometers; Elekta Neuromag). The signals were sampled at a rate of 1000 Hz and online band-pass filtered between 0.03 and 300 Hz. The raw MEG data were visually inspected for artefacts, de-noised and motion-corrected, and downsampled to 250 Hz. Artefacts arising from eye blinks and heartbeats were removed via independent component analysis. Epochs were generated around each stimulus onset (from 0 to 0.6 s) and visually inspected to eliminate any remaining trials with excessive noise. The task consisted of eight brief (approximately 6 min) blocks, in which 480 stimuli were presented (resulting in a total of 3840 stimulus presentations per session). Each block began with the presentation of a target orientation (drawn at random, without replacement, from the 16 stimulus orientations), displayed centrally as a green line. The stimulus stream consisted of randomly oriented Gabor patches, presented centrally for 100 ms, at an average rate of 650 ms. Stimuli had 16 possible angles (5.625–174.375°, in steps of 11.25°). Participants were instructed to respond whenever a Gabor patch with a matching orientation appeared. Since stimuli were drawn uniformly from the 16 possible orientations, 1/16 of all stimuli were targets. The angles were encoded into two covariates using the sine and cosine functions, plus some Gaussian noise for model inference stability. Each block was cut into three shorter segments, giving participants brief rest periods. During the rest periods, the target orientation was presented again as a reminder. Participants were instructed to respond as quickly and accurately as possible.

Human Connectome Project: resting-state fMRI and behavioural data. The resting-state fMRI and behavioural data are available from the Human Connectome Project (HCP) database at db.humanconnectomeproject.org and described in detail in (Van Essen et al., 2013) and (Smith et al., 2013). Briefly, we here used resting-state fMRI and behavioural data from 1,001 subjects from the HCP S1200 release. The fMRI data were collected in a 3T MRI scanner at 2

mm isotropic spatial resolution and a repetition time (TR) of 0.72 seconds over four separate scanning sessions of 14 min. 33 sec. each, which we here concatenated, resulting in 4,800 time points per subject. We used time series in the groupICA50 parcellation. For the predictions, we deconfounded for sex and head motion.

HMM configuration. In all the examples, HMMs were trained for 100 cycles or until convergence, plus 25 cycles from the initialisation step; five initialisations were run, of which the one with the best free energy was continued. This entire training process was repeated 10 times (with different random initializations); the one with the best (lowest) free energy was kept as the final model.
